# The Characteristics and Nomogram for Primary Lung Papillary Adenocarcinoma

**DOI:** 10.1515/med-2020-0014

**Published:** 2020-02-20

**Authors:** Yuqian Zhang, Hui Xie, Ziying Zhang, Pengfei Zhang, Peng Chen, Xiang Wang

**Affiliations:** 1 Department of Thoracic Surgery, The Second Xiangya Hospital, Central South University, 410078, Changsha, China; 2 Department of Oncology, The Third Xiangya Hospital, Central South University, No.138.Tongzipo Road, 410013, Changsha, Hunan, China; 3 Department of Urology, Xiangya Hospital, Central South University, Changsha, Hunan, China

**Keywords:** papillary adenocarcinoma, prognosis, SEER, nomogram, lung cancer

## Abstract

**Background:**

Primary pulmonary papillary adenocarcinoma (PA) is a specific and rare subtype of invasive pulmonary adenocarcinoma (ADC). The knowledge concerning the clinicopathologic features and prognosis of patients with primary pulmonary PA has not been clarified because of its rarity.

**Methods:**

The clinical data of a total of 3391 patients with primary pulmonary PA were retrospectively analyzed to confirm their clinical characteristics and factors influencing prognosis and were in comparison with 3236 patients with non- PA pulmonary adenocarcinoma. All patients were histologically diagnosed between 1988 and 2015 in The Surveillance Epidemiology and End Results (SEER) database. A nomogram with satisfactory predictive performance was established to visually predict long-term survival of these patients.

**Results and conclusion:**

Collectively, primary pulmonary PA is a rare pathological cancer and its prognosis is analogous to that of non-PA pulmonary adenocarcinoma. Older age, larger lesions, distant metastases, lymph node invasion, and poor pathological differentiation are correlative with unacceptable prognosis. Surgical intervention is conducive to reaping favorable prognosis. Unfortunately, radiotherapy or chemotherapy results of no significant effects on patient survival. In our study, a nomogram with prognostic function is formulated to confer individual prediction of overall survival (OS).

## 1 Introduction

Pulmonary carcinoma has multifarious subtypes based on histological pattern and ranks first in both neoplasm incidence and cancer mortality globally [1]. The investigation progress in recent years in the area of lung adenocarcinoma (ADC) has facilitated the occurrence of the 2015 WHO classification of primary lung adenocarcinomas. This WHO classification is dependent on a semi-quantitative evaluation of particular histomorphological growth patterns of invasive adenocarcinoma, with each classified as lepidic, acinar, papillary, micropapillary, or solid predominance. Among them, primary pulmonary papillary adenocarcinoma (PA), also known as papillary-predominant adenocarcinoma (PPA) is a specific and infrequent subtype of invasive adenocarcinoma with a peak incidence ranging from 50 to 60 years old [2]. Primary pulmonary PA accounts for 0.84% among lung cancer [3] and is prone to non-smokers [2]. The patients with pulmonary PA are devoid of extremely specific clinical symptoms such as cough, phlegm, fever, and failure to antibiotic therapy for pneumonia in the early stage [4]. The unique histopathological profile of pulmonary PA is pathologically characterized by the papillary development of cuboidal to columnar cells along with the growth of a fibrovascular core [5]. Radiologically, it primarily exhibits ambiguous pulmonary nodules and is potentially confused with atypical infections [4, 6]. Therefore, early detection is incidental to conventional chest radiographs or CT scans. PA has a distinct immunohistochemistry profile that has prognostic implications.

Due to its rarity, the bulk of studies on primary pulmonary PA have only focused on case reports or serial studies from small institutions. Thus, the demographic and clinicopathological characteristics as well as factors affecting OS, which usually are based on a large-scale patient population, have not been clearly documented. In this retrospective study, the clinical data of total of 3391 patients with primary pulmonary PA were retrospectively analyzed to confirm their clinical characteristics and factors influencing prognosis. The clinical characteristics and OS were summarizing to search for key factors affecting the prognosis of this disease. Simultaneously, A prognostic nomogram of a statistical model with predictive characteristics through calculating a numerical probability of a clinical occurrence is established to help clinicians to individually predict long-term survival of such patients.

## 2 Materials and methods

### 2.1 Participants

This study is approved by, The Second XiangYa Hospital of Central South University. Patient information for this study was obtained from data registered in the U.S. SEER database. The SEER project, encouraged by the National Cancer Institute, collected clinical data from 18 population-based Cancer registries across the United States, including cancer incidence and survival rates, covering about 28 percent of the U.S. population. All patients diagnosed with papillary adenocarcinoma according to ICD-0-3/WHO 2008 (ICD-0-3:8260/3_，_pulmonary carcinoma, papillary type) ranging from 1988 to 2015 were selected from the SEER database. Site record was set to “lung and branchus”, multiple primary fields were set to “one primary only”. Exclusion criteria were set as: patients younger than 18 years old; patients with only autopsy results; and patients with only death certificates. The final enrollment number was 3391. Demographic and clinicopathological characteristics including age, sex, race, lateral position, primary location, pathological differentiation, lesion size (T stage), lymph node metastasis (N stage), remote metastasis (M stage), total tumor stage, whether surgery, radiotherapy or chemotherapy was administered and collected for each patient. The SEER database also reported cancer-specific survival, which defined as the time between diagnosis and death from the cancer or the last follow-up.

### 2.2 Statistical analysis

The Kaplan-Meier was applied for estimating the survival probability, and the log-rank test was utilized to estimate the significant difference in OS stratification among covariates. Cox proportional hazard model was also used to evaluate the relationship between clinicopathological features and OS. HR and 95% CI were estimated via univariate and multivariate models. Independent prognostic factors were determined by multivariate analysis, and only variables significantly related to survival were included in univariate Cox analysis. A prognostic nomogram using all-important independent indicators of OS was generated by our group to predict the survival and prognosis of patients with pulmonary papillary adenocarcinoma and to determine which independent risk factors exert the greatest impact on patients’ prognosis. Statistical analysis was performed using R 3.5.3, and P<0.05 was considered to be statistically significant.

**Ethical statement**: All data downloaded and analyzed were approved by SEER program.

## 3 Results

### 3.1 Patient characteristics

Table 1 shows the demographic and clinicopathological characteristics of the total of 3391 eligible patients enrolled in our study. The age of patient at initial diagnosis was 66 years (from 53 to 77 years). There were more female patients than male patients (53.0% vs. 47%) and the tumor was prone to occur in the right upper lobe of the lung. The majority of cases was graded moderately (grade II, 31.6%) at pathological differentiation; 40% patients with tumor stage T1; 60.2% patients without distant metastasis (M0), and 44.6% patients without lymph node invasion. 1663 (49% of all 3391) patients were performed by surgery intervention and 1289 (38% of all 3391) patients received lobectomy or bilobectomy. Data also shows that chemotherapy was performed for 40.6% of cases, and 30.0% of patients received radiotherapy. Data analysis also shows that patients with radiotherapy or chemotherapy therapy were characterized by larger tumor size, more metastatic lesions with lymph node invasion and advanced tumor stage in comparison to those without radiotherapy or chemotherapy therapy (Supplementary Table S1 and Supplementary Table S2).

**Table 1:** Characteristics of patients with papillary adenocarcinoma

Characteristics	Total	Percent
Age(Mean±SD)	65.89±11.38	
Gender		
Female	1796	53.0%
Male	1595	47.0%
Race		
White	2612	77.0%
Black	344	10.1%
Other	430	12.7%
Unknown	5	0.1%
Marital status		
Married	1923	56.7%
Single	1346	39.7%
Unknown	122	3.6%
Laterality		
Right	1885	55.6%
Left	1298	38.3%
Bilateral	62	1.8%
Unknown	146	4.3%
Lobe		
Main Bronchus	54	1.6%
Upper	1473	43.4%
Middle	178	5.2%
Lower	1122	33.1%
Overlapping lesion of lung	45	1.3%
Unknown	519	15.3%
Grade		
Well; I	558	16.5%
Moderately; II	1071	31.6%
Poorly;III	337	9.9%
Undifferentiated; IV	28	0.8%
Unknown	1397	41.2%
Tumor stage		
T1	1356	40.0%
T2	746	22.0%
T3	269	7.9%
T4	173	5.1%
TX	847	25.0%
Node status		
N0	1512	44.6%
N1	311	9.2%
N2	748	22.1%
N3	241	7.1%
NX	579	17.1%
Metastasis status		
M0	2041	60.2%
M1	1220	36.0%
MX	130	3.8%
Summary stage		
Localized	949	28.0%
Regional	832	24.5%
Distance	1521	44.9%
Unknown	89	2.6%
TNM		
I	892	26.3%
II	346	10.2%
III	427	12.6%
IV	1220	36.0%
Unknown	506	14.9%
Surgery		
Yes	1663	49.0%
No	1722	50.8%
Unknown	6	0.2%
Surgery type		
Lobectomy/Bilobectomy	1289	38.0%
Partial resection	76	2.2%
Pneumonectomy	278	8.2%
Surgery, NOS	20	0.6%
Radiation		
No	2375	70.0%
Yes	1016	30.0%
Chemotherapy		
No	2014	59.4%
Yes	1377	40.6%

Abbreviation: SD, standard deviation

### 3.2 Overall survival and prognostic factors

**Figure 1:**
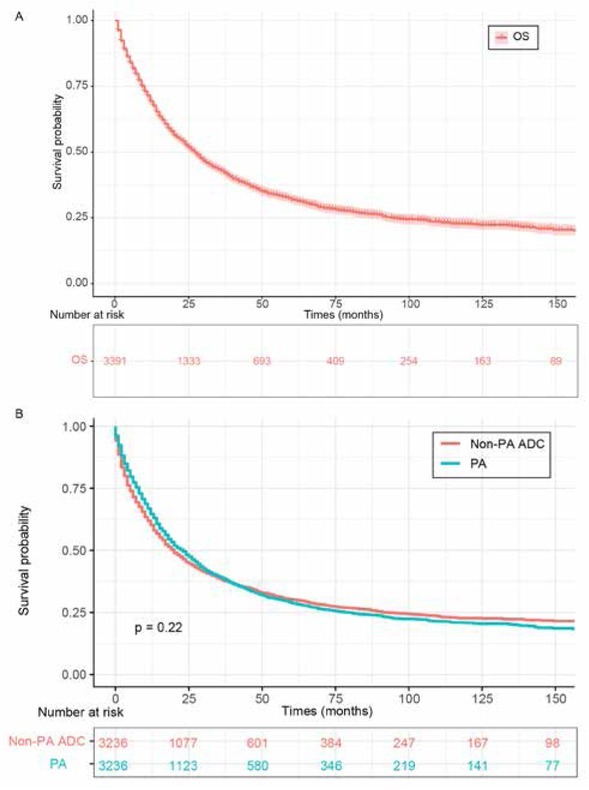
OS for primary pulmonary PA patients. (A) total OS for pulmonary PA patients. (B) OS comparison between primary pulmonary PA and matched non-PA lung adenocarcinoma. Abbreviations: OS, overall survival; PA, papillary adenocarcinoma.

The mean OS of the total 3391 pulmonary PA patients was 32.6 months (95% CI 31.2-33.9 months) (Figure 1A and Table 2). The overall 3-, 5- and 10-year survival rate for patients with primary pulmonary PA were 43%, 32%, and 22%, respectively (Supplementary Table S3). 3236 pulmonary PA patients were matched with 3236 non-PA pulmonary adenocarcinoma patients (1:1), which were also pathologically confirmed ranging from 1988 to 2015 in the SEER database, to figure out the prognostic difference between these two groups, and here 155 patients in all 3391 had been discarded during matching. Our study found no significant differences in clinical features following propensity-score matching (PSM) analysis (Supplementary Table S4). And there is also no significant difference in clinical prognosis between pulmonary PA patients and counterparts with non-PA adenocarcinoma (Figure 1B).

**Table 2:** Overall survival stratified by clinical characteristics and univariate Cox proportional hazard analyses for patients

			Univariate analysis	
Variables				
	Mean survival (months)	95% CI	HR(95%CI)	P-value
Total	32.6	(31.2-33.9)		
Age				
<=78	33.9	(32.4-35.5)	Ref	
>78	23.2	(20.5-25.8)	1.29(1.13-1.43)	<0.001
Gender				
Female	21.8	(20.33-23.31)	Ref	
Male	20.9	(19.35-22.52)	1.06(0.97-1.15)	0.202
Race				
White	21.6	(20.37-22.89)	Ref	
Black	17.3	(14.33-20.19)	1.13(0.97-1.31)	0.110
Other	23.3	(20.35-26.29)	0.92(0.8-1.05)	0.200
Marital status				
Married	22.4	(20.96-23.86)	Ref	
Single	20.0	(18.3-21.75)	1.06(0.97-1.16)	0.232
Laterality				
Right	21.9	(20.43-23.3)	Ref	
Left	22.3	(20.35-24.16)	1.07(0.98-1.17)	0.153
Bilateral	15.9	(9.83-22.04)	2.49(1.87-3.32)	<0.0001
Lobe				
Upper	12.3	(8.01-16.57)	Ref	
Middle	22.0	(20.41-23.69)	0.75(0.6-0.93)	0.011
Lower	29.7	(21.3-38.04)	0.92(0.83-1.02)	0.956
Main bronchus	24.9	(22.79-27.1)	2.19(1.62-2.95)	<0.001
Overlapping lesion of lung	20.0	(12-28)	1.33(0.93-1.89)	0.113
Grade				
Well; I	30.6	(26.82-34.48)	Ref	
Moderately; II	28.0	(25.5-30.5)	1.14(0.98-1.32)	0.098
Poorly; III	21.1	(17.94-24.32)	1.94(1.62-2.32)	<0.001
Undifferentiated; IV	21.0	(10.32-31.68)	1.47(0.9-2.41)	0.122
Node status				
N0	29.4	(26.87-31.9)	Ref	
N1-3	19.5	(18.16-20.89)	2.93(2.64-3.25)	<0.001
Metastasis status				
M0	28.9	(27.05-30.77)	Ref	
M1	13.8	(12.77-14.74)	3.62(3.29-3.97)	<0.001
Tumor size				
<=38mm	41.7	(39.4-44.1)	Ref	
>38mm	29.6	(27.2-31.9)	1.88(1.69-2.10)	<0.001
Surgery				
Yes	35.8	(33.38-38.23)	Ref	
No	14.3	(13.44-15.21)	4.77(4.33-5.26)	<0.001
Surgery type				
Lobectomy/Bilobectomy	52.8	(49.9-55.6)	Ref	
Partial resection	43.7	(32.1-55.4)	1.83(1.33-2.5)	<0.001
Pneumonectomy	39.4	(33.9-44.8)	1.8(1.49-2.17)	<0.001
Radiation				
Yes	22.8	(21.3-24.22)	Ref	
No	19.1	(17.57-20.71)	0.52(0.47-0.57)	<0.001
Chemotherapy				
No	22.5	(20.77-24.14)	Ref	
Yes	20.3	(18.95-21.66)	1.8(1.64-1.96)	<0.001

Abbreviation: 95% CI, 95% Confidence interval

**Figure 2:**
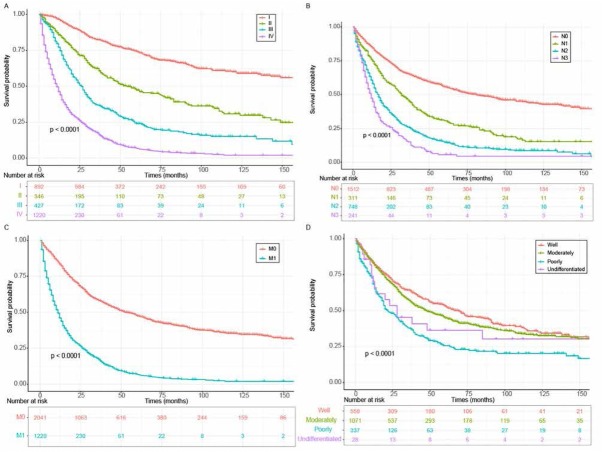
OS for primary pulmonary PA patients classified by (A) TNM stages. (B) lymph node involvement. (C) distant metastases. (D) pathological differentiation grade. Abbreviations: OS, overall survival; PA, papillary adenocarcinoma.

Patients with more advanced disease stages were endowed with unsatisfactory clinical outcome. Indeed, the 3-, 5-, and 10- year survival rates for patients with TNM I were 82%_，_74%_，_60% respectively; In TNM II patients, the 3-, 5-, and 10- year survival rates decreased to 62%, 47%, 30% respectively and the rates decreased further in TNM III patients to 37%, 24%, 15%, respectively. In TNM stage IV patients, the 3-, 5-, and 10- years survival rates were 17%, 7% and 2% (Supplementary Table S3 and Figure 2A). Our study also found that the tumor Summary lesion stage plays a role in survival rates. The 3-year, 5-,year, and 10-year survival rates for patients with localized lesion stage were 77%, 69%, and 7% respectively, and the rates dropped dramatically to 52%, 37%, and 22% respectively in patients with regional lesions. In patients with distance lesion stage the 3-year, 5-year and 10-year survival rates dropped even more to 17%, 7%, and 3% respectively (Supplementary Table S3 and Supplementary Figure S1A). Lymph node status also plays a role. Patients with any lymph node involvement (N1-3) and remotely metastatic lesions (M1) in pulmonary PA patients were associated with undesirable prognosis (Figure 2B and Figure 2C). We also found that the prognosis of pulmonary PA patients with well or moderately pathologically differentiated pulmonary tumors were superior to those with poorly differentiated or undifferentiated tumors (Figure 2D). Intriguingly, patients with bilateral tumor located in the main bronchus correlated with inferior clinical outcomes (Supplementary Figure S1B and Supplementary Figure S1C). While gender of patient appeared to show no effect on prognosis (Supplementary Figure 1D).

**Figure 3:**
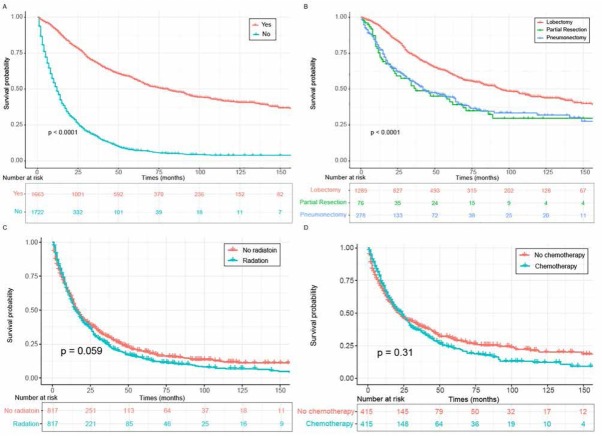
OS for primary pulmonary PA patients classified by (A) surgery. (B) different surgery type. (C) radiation after PSM analysis. (D) chemotherapy after PSM analysis. Abbreviations: OS, overall survival; PA, papillary adenocarcinoma; PSM, propensity-score matching.

We further found that surgical treatment could provide an expressively prolonged survival time for pulmonary PA patients (35.8 months vs 14.3 months) (Figure 3A). With regard to surgical categories, patients who received lobectomy or bilobectomy had significantly superior outcomes than those managed by partial resection or pneumonectomy (P < 0.01 for both) (Figure 3B). Notably, radiotherapy was instrumental in extending survival time while chemotherapy exerted a detrimental effect on prognosis without PSM analysis (P < 0.0001 for both) (Supplementary Figure S2A and Supplementary Figure S2B). However, with PSM analysis to exclude certain confounding factors (Supplementary Table S1 and Supplementary Table S2), our study demonstrated that radiation or chemotherapy imposed no effect on OS (Figure 3C and Figure 3D).

### 3.3 Univariate and multivariate Cox proportional hazard analyses

According to the univariate analysis of prognostic factors (Table 2), patients with older age; bilateral and larger lesions located in the main bronchus; poor pathological differentiation; lymph node invasion and remote metastasis; as well as chemotherapy subjected with undesirable prognosis. Inversely, surgical intervention and radiation therapy were conducive to improving OS (P<0.05 for all). Multivariate Cox analysis including above statistically significant univariate further showed that older age at primary diagnoses; poor pathological differentiation; larger lesion size; lymph node metastasis and distant metastatic lesions; were independent prognostic factors for patients’ worsening survival. Surgical intervention was independent protective factors for boosted survival time (Table 3).

**Table 3:** Multivariate Cox proportional hazard analyses of clinical features for overall survival rates in patients with primary pulmonary PA

	Multivartiate anlysis	
Variables		
	HR[95%CI]	P-value
Age		
<=78	Ref	
>78	1.43(1.25-1.63)	<0.001
Grade		
Well; I	Ref	
Moderately; II	1.2(1.03-1.4)	0.021
Poorly;III	1.51(1.26-1.82)	<0.001
Undifferentiated; IV	0.8(0.48-1.31)	0.368
Node status		
N0	Ref	
N1-3	1.87(1.67-2.1)	<0.001
Metastasis status		
M0	Ref	
M1	1.6(1.42-1.79)	<0.001
Tumor size		
<=38mm	Ref	
>38mm	1.45(1.3-1.62)	<0.001
Surgery		
Yes	Ref	
No	2.53(2.19-2.91)	<0.001
Radiation		
Yes	Ref	
No	0.83(0.75-0.91)	<0.001
Chemotherapy		
No	Ref	
Yes	1.05(0.95-1.16)	0.316

Abbreviation: HR: hazard ratio; 95% CI, 95% Confidence interval, PA, papillary adenocarcinoma.

**Figure 4:**
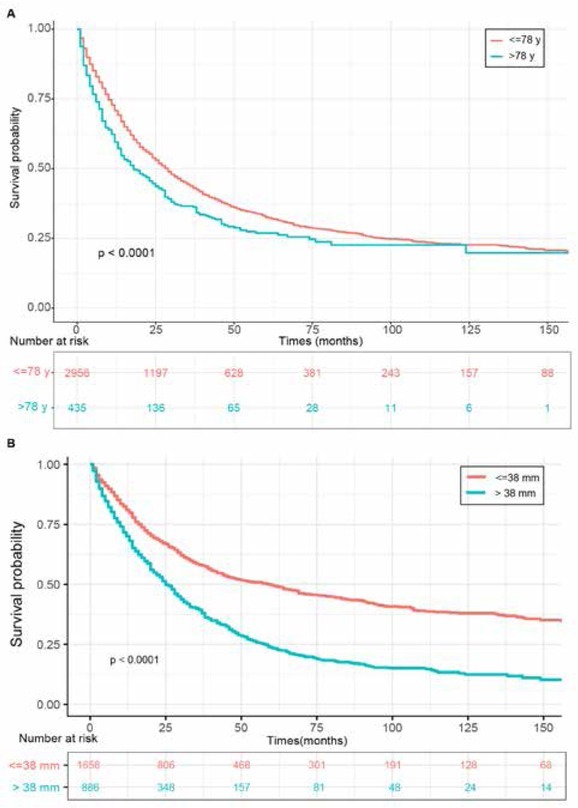
OS for primary pulmonary PA patients classified by (A) age. (B) tumor size. Abbreviations: OS, overall survival; PA, papillary adenocarcinoma.

Furthermore, the X-tile program showed that older patients with larger tumor size were associated with dis-advantageous clinical outcomes (Figure 4A and Figure 4B). 78 years and 38 mm were the optimal cut-points to estimate prognosis for age at diagnosis and tumor size, respectively (Supplementary Figure S3). All included pulmonary PA patients could be stratified into two groups which represented significant differences in age- and size-associated OS, respectively.

### 3.4 Prognostic nomogram for primary pulmonary PA

**Figure 5:**
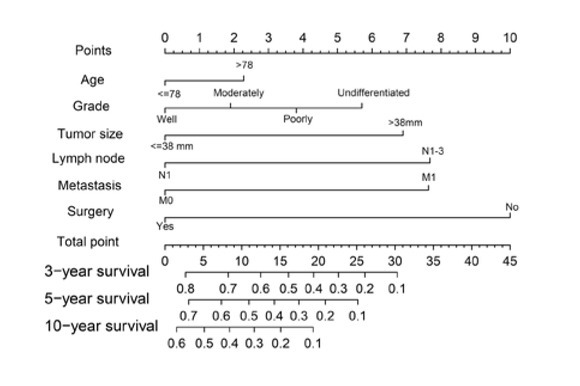
Novel nomogram estimated by clinical characteristics for the overall 3-, 5-, and 10-year survival rate in patients with primary pulmonary PA. Abbreviations: PA, papillary adenocarcinoma.

Six independent prognostic factors were determined and included in the nomogram model (Figure 5). This nomogram with a C-index of 0.747 showed that surgery exerted the greatest effect on prognosis, followed by lymph node invasion and remote metastasis. The impact of tumor size and pathological differentiation degree on prognosis was relatively moderate, while age had the least effect on prognosis. Each of these variables was assigned to a specific score. By accumulating the total score and positioning it on the total score table, it was simple to draw a line to conclude the estimate of survival probability at each score point. The calibration plots for the OS probability of 3-, 5-, or 10- years in primary pulmonary PA patient cohort were endowed with a favorable conformance between the nomogram prediction and actual surveillance (Supplementary Figure S4).

## 4 Discussion

Primary pulmonary PA is an extremely rare subtype of adenocarcinoma. The majority of studies on pulmonary PA is composed of small series or individual case reports because of its rarity. In this report, we studied the clinical characteristics of 3,391 patients with pulmonary PA and determined the variables affecting OS through utilizing SEER database data from 1988 to 2015, which would be instrumental in guiding us to develop more rational management for pulmonary PA patients.

In the prior sporadic cases, the overwhelming majority of patients with pulmonary PA were diagnosed at approximately 65 years-of-age, with a proneness to the female, which was roughly consistent with another study [7]. The majority of lesions had an occurrence tendency in the upper lobe and such malignant lesions were relatively small. Indeed, a study showed tumor size (diameter >29 mm) was correlated with more aggressive PA [8]. In the current study, the majority of patients had larger lesion sizes than recorded by the above group [9].

A cohort study based on analyzing 226 Chinese patients with PPA, the 5-year survival rate was 61.50% for subtypes PPA [2]. Another Japanese study retrospectively analyzed 2004 patients with PPA and further concluded that the 5-year overall survival rates of such tumor was 72.9% [9]. In our study, we revealed that such patients’ 5-year survival rate was 32%, which was relatively lower than above study. This discrepancy is potentially attributable to different ethnicity, genetic background, or sample size. We also found that the prognosis of PA was analogous to that of non-PA ADC. The majority of additional studies concluded that in invasive ADC, patients with lepidic pre-dominant adenocarcinoma (LPA) were endowed with the best survival, followed by acinar predominant adenocarcinoma (APA) and papillary predominant adenocarcinoma (PPA), while patients with solid predominant adenocarcinoma (SPA) and mucin production adenocarcinoma (MPA) showed the worst survival rates [8, 10, 11, 12 13]. Our present study found 36.0% of patients were in the condition of TNM IV and pathological characteristics exerted a remarkable effect in determining OS of pulmonary PA patients, which was similar to additional studies[14]. Worse pathological differentiation degree of tumor cell is prominently correlated with unsatisfactory clinical outcome [2]. Specifically, in this cohort, pulmonary PA subjects with poor pathological differentiation had merely a mean OS of 21.1 months while patients with well pathologically differentiated tumor had significant longer 30.6 months. Besides, another one of the valuable prognostic factors in primary pulmonary PA was lymph node involvement [14]. Ikuo et al revealed the higher occult nodal metastasis rate in PPA subtype (17/154, 11.0%) and SPA subtype (2/7, 28.6%) (both P=0.001) in comparison with additional subtypes through retrospectively analyzing 237 patients with peripheral clinical stage I lung adenocarcinoma [15]. In our current study, 38.4% of pulmonary PA exhibited positive lymph node invasion. In the same vein, subjects with larger lesions and distant metastases were endowed with more undesirable survival time. Accordingly, early detection is potentially of great essence to acquire first-rank clinical outcomes for pulmonary PA patients.

Surgical intervention was considered as the optimal treatment and contributed to an excellent prognosis in such patients [16]. In terms of operative mode, we revealed that 38.0% of patients were managed by lobectomy or bilobectomy and could reap the optimal therapeutic effect. Indeed, a retrospective study also demonstrated that 84.6% of patients received lobectomy, followed by segmentectomy/wedge resection and pneumonectomy [9]. It is worth mentioning that pneumonectomy conferred a much more undesirable clinical prognosis than lobectomy or resection, which was potentially attributable to the extensive loss of pulmonary function [17]. The impact of chemotherapy on patients with lung papillary adenocarcinoma is still controversial. Our study showed that chemotherapy had no significant benefit for pulmonary PA patients. Similarly, Chen et al also revealed that in the PPA subgroup, there was no statistically significant benefit from adjuvant chemotherapy (ACT) for both disease-free survivals (DFS) [18]. Nevertheless, a contradictory result was elicited in two independent sporadic case reports, which described that two patients with lung papillary adenocarcinoma characterized by paraneoplastic syndrome were both performed for cancer-directed pulmonary lobectomy and postoperative adjuvant chemotherapy, thus representing imaging and laboratory indicators during long-term follow-up. These results indicate the effectiveness and significance of surgical intervention in combination with chemotherapy [3, 19]. A study revealed that patients with PPA also benefited from postoperative radiotherapy (PORT) with an increase in OS (HR: 0.350, 95% CI: 0.126 to 0.972, P = 0.033) [20].

In the light of current literature review, our study developed the first nomogram to predict the survival and prognosis of patients with pulmonary PA based on SEER database. Nevertheless, our nomogram did not include other clinical information, including pivotal clinical symptoms, imaging presentations, and comprehensive laboratory indicators such as serum tumor markers [15]. More importantly, accumulating clinical studies revealed the identification of such tumors with alterations in genes such as ALK [21], epidermal growth factor receptor (EGFR) [22, 23, 24, 25], which was momentous to guide tyrosine kinase inhibitors (TKIs) therapy. As an example, approximately 71% of Asiatic patients with PPA harbored EGFR mutation [26] and EGFR‐TKIs was used to lengthen progression‐ free survival in patients with an EGFR gene mutation and was introduced as first‐line therapy in these patients [27]. Moreover, rearrangements of ALK highlighted disease recurrence [23]. With the advent of immune checkpoint inhibitors, PD-L1 protein expression was considered as a well-characterized predictive biomarker for immunotherapy of PPA patients [28]. Therefore, further efforts are warranted to make in the collection of such indicators, thus improving this model to make a more comprehensive prognostic prediction of patients with pulmonary PA.

Notably, several limitations in this study should be discussed. Firstly, the retrospective property of such a study is devoid of prospective studies or randomized controlled trials. We only conducted a retrospective investigation based on the existing patient information in the database. Secondly, the information about the patient’s treatment effect, recurrence, and whether there are complications or not cannot be obtained from the database. Thirdly, the SEER database only provides tumors occurring among the US population, and other areas with high incidences of PA cannot be obtained for more rigorous and comprehensive analysis.

## 5 Conclusion

Although pulmonary PA is rare, we utilized a population-based approach to roughly stratify the prognosis based on identifying variables. The study found that older age, poor pathological differentiation, larger tumor size, lymph node involvement, and distant metastasis were all independent risk factors for pulmonary papillary adenocarcinoma. Early surgical treatments could prolong the survival time of patients, in which lobectomy was the most effective. A new nomogram was also developed to estimate the survival and prognosis of patients with this disease. For this rare subtype of lung cancer, doctors can accurately estimate a patient’s chances of survival. These data will be potentially instrumental in future management and prospective studies of pulmonary PA patients.

## Abbreviations

PA: papillary adenocarcinoma;
ADC: adenocarcinoma;
SEER: Surveillance, Epidemiology and End Results;
OS: overall survival;
PPA: papillary predominant adenocarcinoma;
EGFR: epidermal growth factor receptor;
LPA: Lepidic predominant adenocarcinoma;
APA: acinar predominant adenocarcinoma;
SPA: solid predominant adenocarcinoma;
MPA: mucin production adenocarcinoma;
PSM: propensity-score matching;
C-index: concordance index;
ACT: adjuvant chemotherapy;
DFS: disease-free survival;
TKIs: tyrosine kinase inhibitors;
HR: hazard ratio.

## References

[1] Siegel RL, Miller KD, Jemal A. (2019). Cancer statistics, 2019. CA Cancer J Clin.

[2] Dong Y, Li Y, Jin B, Zhang J, Shao J, Peng H (2017). Pathologic subtype-defined prognosis is dependent on both tumor stage and status of oncogenic driver mutations in lung adenocarcinoma. Oncotarget.

[3] Xing L, Wang H, Qu W, Fang F, Dong QE, Shao Z (2014). Lung papillary adenocarcinoma complicated with paraneoplastic autoimmune hemolytic anemia: A case report. Thorac Cancer.

[4] Gupta A, Palkar A, Narwal P. (2018). Papillary lung adenocarcinoma with psammomatous calcifications. Respir Med Case Rep.

[5] Aida S, Shimazaki H, Sato K, Sato M, Deguchi H, Ozeki Y (2004). Prognostic analysis of pulmonary adenocarcinoma subclassification with special consideration of papillary and bronchioloalveolar types. Histopathology.

[6] Austin JH, Garg K, Aberle D, Yankelevitz D, Kuriyama K, Lee HJ (2013). Radiologic implications of the 2011 classification of adenocarcinoma of the lung. Radiology.

[7] Jian Z, Tomizawa Y, Yanagitani N, Iijima H, Sano T, Nakajima T (2005). Papillary adenocarcinoma of the lung is a more advanced adenocarcinoma than bronchioloalveolar carcinoma that is composed of two distinct histological subtypes. Pathol Int.

[8] Russell PA, Wainer Z, Wright GM, Daniels M, Conron M, Williams RA (2011). Does lung adenocarcinoma subtype predict patient survival?: A clinicopathologic study based on the new International Association for the Study of Lung Cancer/ American Thoracic Society/European Respiratory Society international multidisciplinary lung adenocarcinoma classification. J Thorac Oncol.

[9] Sakurai H, Asamura H, Miyaoka E, Yoshino I, Fujii Y, Nakanishi Y (2014). Differences in the prognosis of resected lung adenocarcinoma according to the histological subtype: a retrospective analysis of Japanese lung cancer registry data. Eur J Cardiothorac Surg.

[10] Nakamura H, Takagi M (2015). Clinical impact of the new IASLC/ATS/ ERS lung adenocarcinoma classification for chest surgeons. Surg Today.

[11] Song Z, Zhu H, Guo Z, Wu W, Sun W, Zhang Y (2013). Prognostic value of the IASLC/ATS/ERS classification in stage I lung adenocarcinoma patients--based on a hospital study in China. Eur J Surg Oncol.

[12] Gu J, Lu C, Guo J, Chen L, Chu Y, Ji Y (2013). Prognostic significance of the IASLC/ATS/ERS classification in Chinese patients-A single institution retrospective study of 292 lung adenocarcinoma. J Surg Oncol.

[13] Yu W, Zhao Q, Xia C, Dong M, Liu J, Li X (2019). Validation of stage groupings in the eighth edition of the tumor node metastasis classification for lung adenocarcinoma. Thorac Cancer.

[14] Park JK, Kim JJ, Moon SW, Lee KY (2017). Lymph node involvement according to lung adenocarcinoma subtypes: lymph node involvement is influenced by lung adenocarcinoma subtypes. J Thorac Dis.

[15] Song CY, Kimura D, Sakai T, Tsushima T, Fukuda I (2019). Novel approach for predicting occult lymph node metastasis in peripheral clinical stage I lung adenocarcinoma. J Thorac Dis.

[16] Duann CW, Hung JJ, Hsu PK, Huang CS, Hsieh CC, Hsu HS (2013). Surgical outcomes in lung cancer presenting as ground-glass opacities of 3 cm or less: a review of 5 years’ experience. J Chin Med Assoc.

[17] Huang Y, Yang X, Lu T, Li M, Zhao M, Yang X (2018). Assessment of the prognostic factors in patients with pulmonary carcinoid tumor: a population-based study. Cancer Med.

[18] Luo J, Huang Q, Wang R, Han B, Zhang J, Zhao H (2016). Prognostic and predictive value of the novel classification of lung adenocarcinoma in patients with stage IB. J Cancer Res Clin Oncol.

[19] Yu H, Fu R, Wang H, Liu H, Shao Z (2017). Paraneoplastic Evans syndrome in a patient with adenocarcinoma of the lung: A case report. Thorac Cancer.

[20] Yuan C, Tao X, Zheng D, Pan Y, Ye T, Hu H (2019). The lymph node status and histologic subtypes influenced the effect of postoperative radiotherapy on patients with N2 positive IIIA non-small cell lung cancer. J Surg Oncol.

[21] Grosser DS, Zhang H (2019). Histomorphologic features of lung adenocarcinomas exhibiting ALK gene rearrangement. Proc (Bayl Univ Med Cent).

[22] Katayama Y, Kawai S, Miyagawa-Hayashino A, Takemura Y (2019). Multiple primary lung adenocarcinomas pre-operatively diagnosed by discordant epidermal growth factor receptor mutations. Respirol Case Rep.

[23] Feng Y, Feng G, Lu X, Qian W, Ye J, Manrique CA (2018). Exploratory analysis of introducing next-generation sequencing-based method to treatment-naive lung cancer patients. J Thorac Dis.

[24] Forest F, Patoir A, Dal-Col P, Da Cruz V, Camy F, Stachowicz ML (2018). Lepidic, Papillary Components and EGFR Mutations are Frequent in Patients With Lung Adenocarcinoma Who are Over 75 Years Old. Appl Immunohistochem Mol Morphol.

[25] Sun F, Xi J, Zhan C, Yang X, Wang L, Shi Y (2018). Ground glass opacities: Imaging, pathology, and gene mutations. J Thorac Cardiovasc Surg.

[26] Chen Z, Liu X, Zhao J, Yang H, Teng X (2014). Correlation of EGFR mutation and histological subtype according to the IASLC/ATS/ERS classification of lung adenocarcinoma. Int J Clin Exp Pathol.

[27] Wu SG, Shih JY (2018). Management of acquired resistance to EGFR TKI-targeted therapy in advanced non-small cell lung cancer. Mol Cancer.

[28] Farkasova A, Tancos V, Kviatkovska Z, Hutka Z, Micak J, Scheerova K Clinicopathological analysis of programmed death-ligand 1 testing in tumor cells of 325 patients with non-small cell lung cancer: Its predictive and potential prognostic value. Cesk Patol.

