# Supplementary material for: The Characteristics and Nomogram for Primary Lung Papillary Adenocarcinoma
Source: Open Med (Wars). 2020 Feb 20;15:92–102. doi: 10.1515/med-2020-0014 (PMC7070103; doi:10.1515/med-2020-0014)
Supplement: Supplementary file 1 [file med-15-092_sm.pdf]

Electronic supplementary material

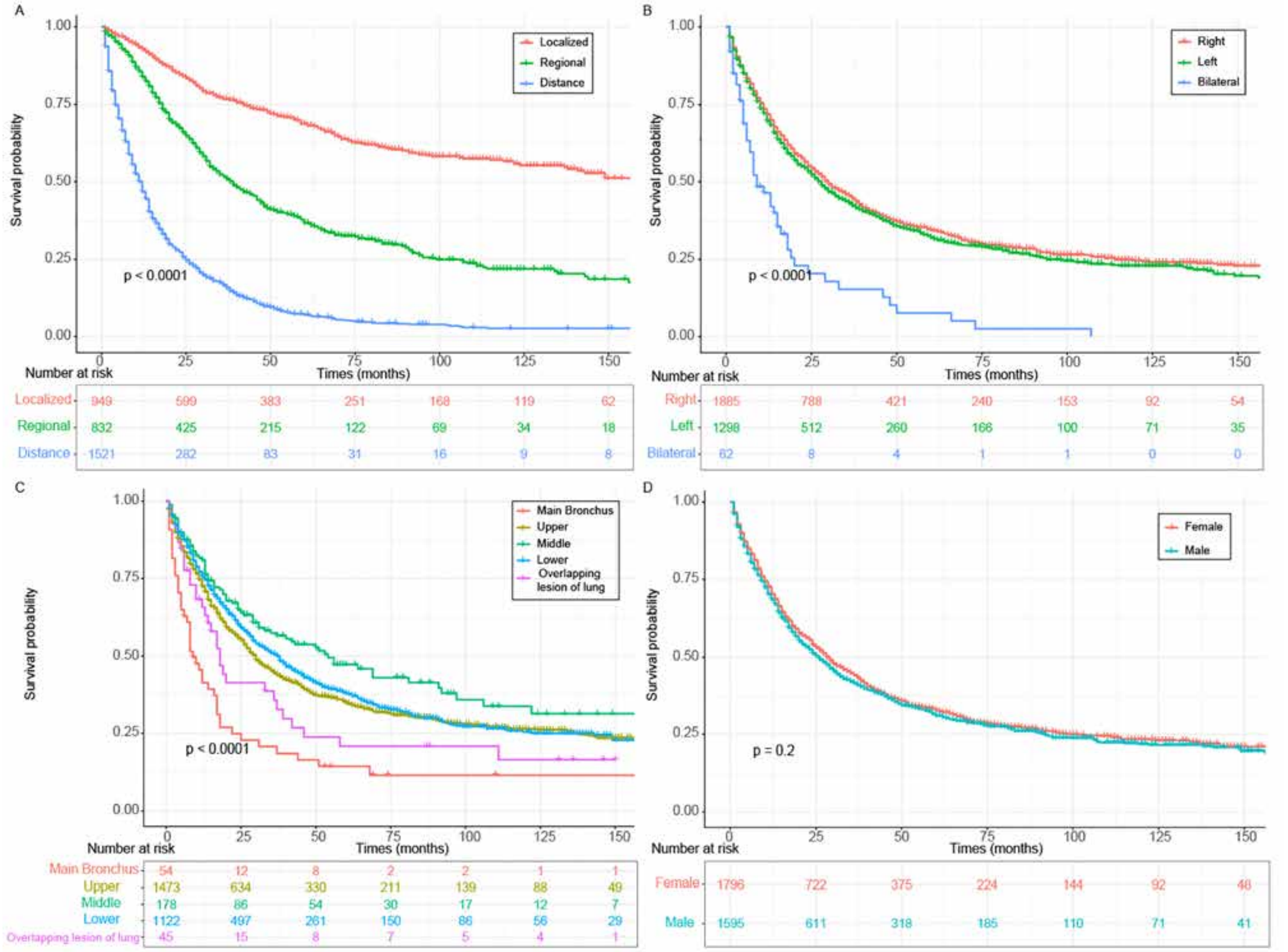

Supplementary Figure S1: OS for primary pulmonary PA patients classified by (A) SEER summary stages. (B) laterality. (C) lobe. (D) sex. Abbreviations: OS, overall survival; PA, papillary adenocarcinoma; SEER, Surveillance, Epidemiology and End Results.

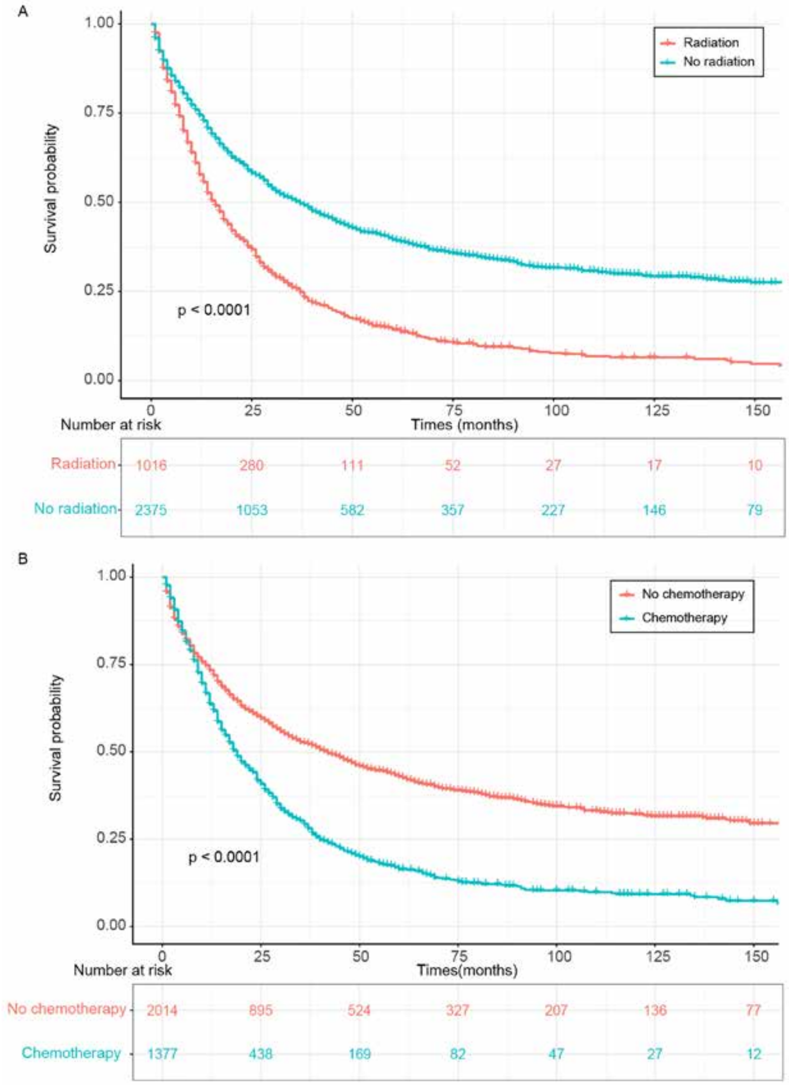

Supplementary Figure S2: OS for primary pulmonary PA patients classified by (A) radiation before PSM analysis. (B) chemotherapy before PSM analysis

Abbreviations: OS, overall survival; PA, papillary adenocarcinoma; PSM, propensity-score matching.

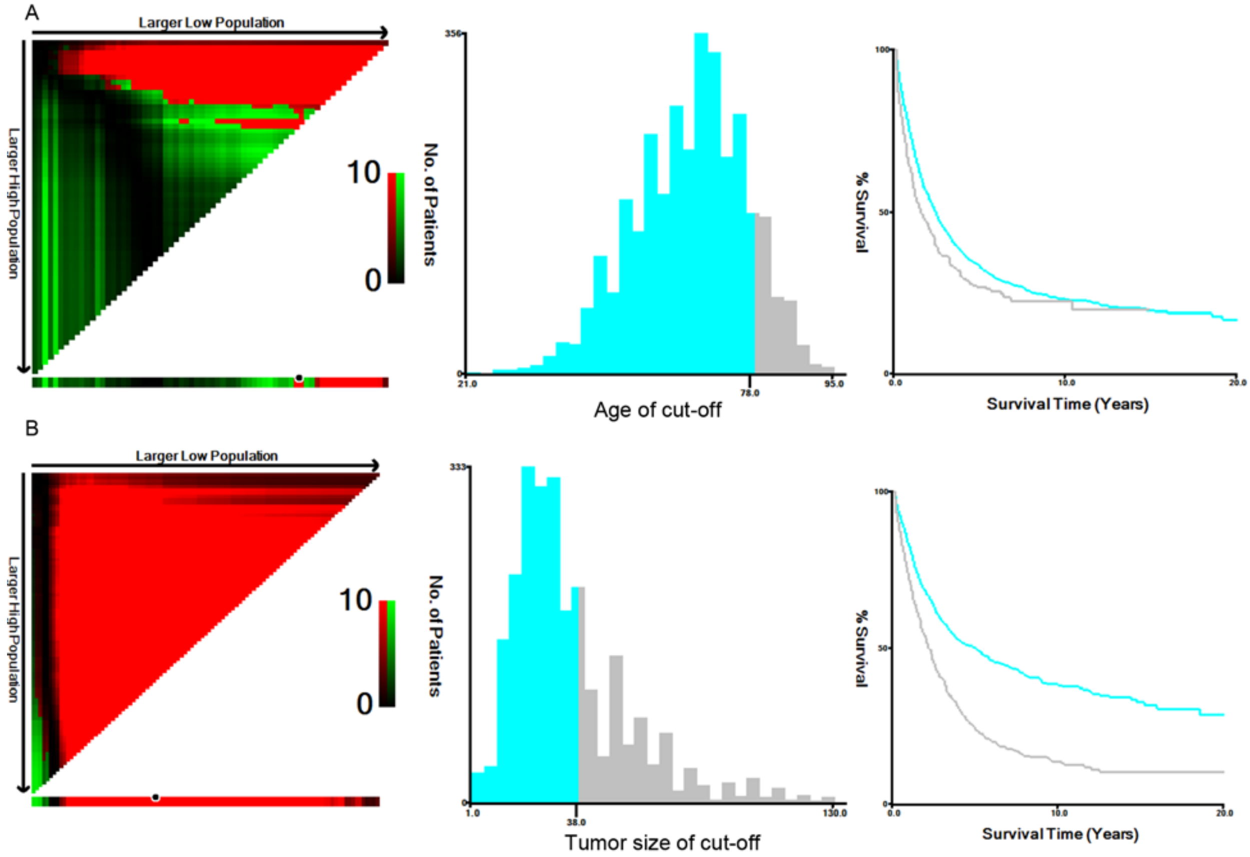

Supplementary Figure S3: The optimal cut-off of tumor size and age in primary pulmonary PA patients.
Abbreviations: PA, papillary adenocarcinoma.

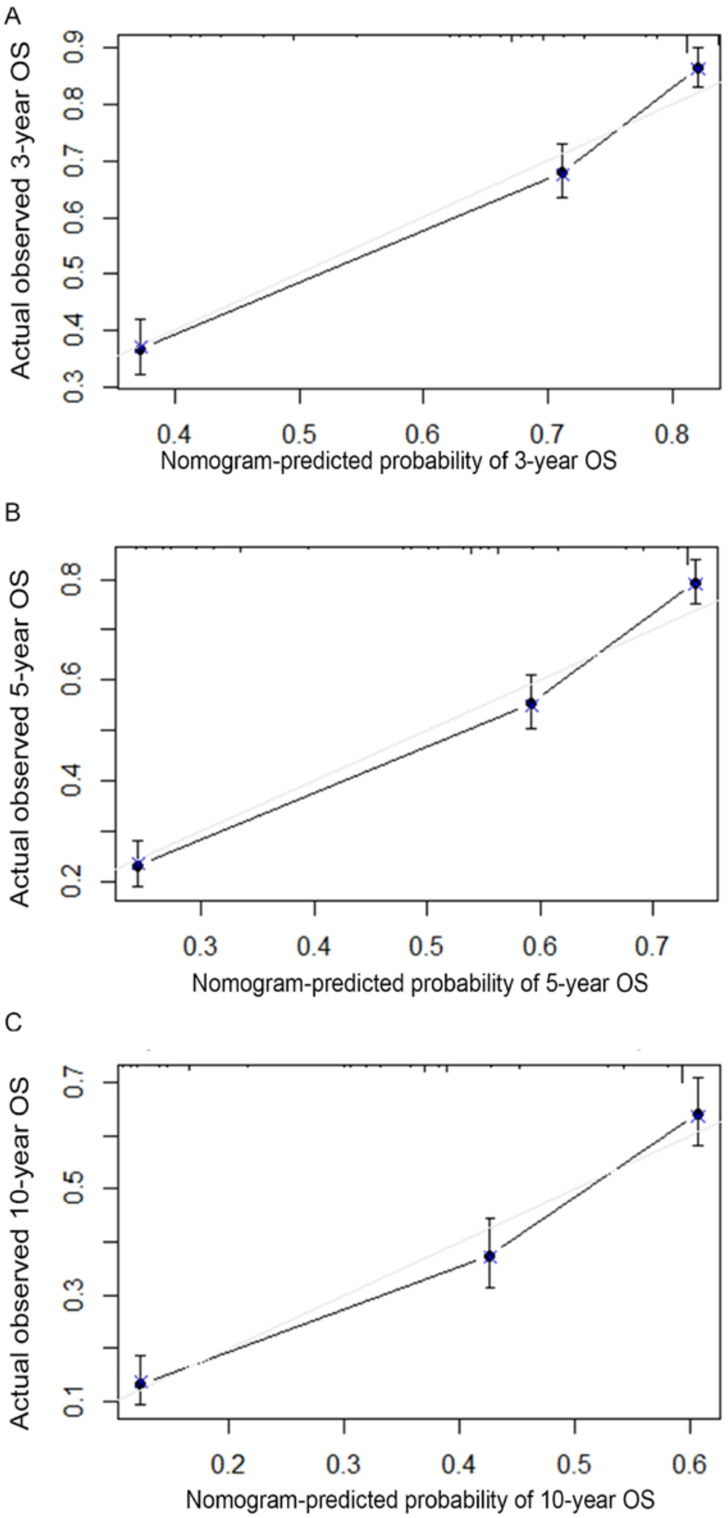

Supplementary Figure S4: The calibration curves to estimate primary pulmonary PA patient survival at (A) 3 years and (B) 5 years, and (C) 10 years.

Abbreviations: PA, papillary adenocarcinoma.

Supplementary Table S1: Patient characteristics by radiation before and after PSM

Variables		No Radiation	Radiation	P value	No Radiation	Radiation	P value
Total		2375	1016		817	817	
Age(Mean±SD)		66.8±11.7	63.7±11.8	<0.001	65.0±11.8	64.6±11.6	0.53
Gender				0.29			0.25
	Female	1244	552		455	432	
	Male	1131	464		362	385	
Race				0.13			0.65
	White	1851	761		617	614	
	Black	223	121		82	95	
	Unknown	301	134		118	108	
Marital status				0.17			0.51
	Married	1323	600		467	465	
	Single	962	384		329	323	
	Unknown	90	32		21	29	
Laterality				<0.001			0.39
	Right	1342	543		423	441	
	Left	916	382		323	305	
	Bilateral	41	21		26	18	
	Unknown	76	70		45	53	
Lobe				<0.001			0.59
	Upper	30	24		21	147	
	Middle	1004	469		350	9	
	Lower	135	43		36	243	
	Main bronchus	826	296		226	38	
	Overlapping lesion of lung	35	10		12	363	
	Unknown	345	174		172	17	
Grade				<0.001			0.13
	Well; I	456	102		100	90	
	Moderately; II	841	230		205	196	
	Poorly;III	217	120		71	103	
	Undifferentiated; IV	19	9		5	7	
	Unknown	842	555		436	421	
Tumor stage				<0.001			0.01
	T1	1040	316		264	245	
	T2	470	276		160	43	
	T3	173	96		68	71	
	T4	126	47		58	219	
	TX	566	281		267	239	
Node status				<0.001			0.46
	N0	1285	227		190	215	
	N1	213	98		101	82	

Variables		No Radiation	Radiation	P value	No Radiation	Radiation	P value
	N2	381	367		253	250	
	N3	127	114		83	80	
	NX	369	210		190	190	
Metastasis status				<0.001			<0.001
	M0	1643	398		403	30	
	M1	637	583		369	447	
	MX	95	35		45	340	
Summary stage				<0.001			0.29
	Localized	878	71		85	69	
	Regional	573	259		209	220	
	Distance	857	664		494	508	
	Unknown	67	22		29	20	
Surgery				<0.001			0.71
	No	1406	257		246	239	
	Yes	964	758		571	578	
Chemotherapy				<0.001			0.58
	No	1613	401		360	371	
	Yes	762	615		457	446	

Abbreviation: PSM: Propensity-score matching; SD, standard deviation

Supplementary Table S2: Patient characteristics by chemotherapy before and after PSM

Variables		No Chemotherapy	Chemotherapy	P value	No Chemotherapy	Chemotherapy	P value
Total		2014	1377		415	415	
Age(Mean±SD)		68.2±10.7	62.6±11.5	<0.001	65.5±11.8	65.8±11.3	0.72
Gender				0.45			0.36
	Female	1056	958		216	185	
	Male	740	637		199	230	
Race				0.007			0.3
	White	1590	1022		307	312	
	Black	196	148		59	43	
	Unknown	228	207		49	60	
Marital status				<0.001			0.06
	Married	1065	858		225	250	
	Single	874	472		180	149	
	Unknown	75	47		10	16	
Laterality				0.006			0.49
	Right	1128	757		244	225	
	Left	783	515		248	161	
	Bilateral	25	37		6	10	
	Unknown	78	68		17	19	
Lobe				<0.001			0.79
	Upper	22	32		5	6	
	Middle	901	572		169	167	
	Lower	118	60		19	25	
	Main bronchus	708	414		143	128	
	Overlapping lesion of lung	21	24		8	10	
	Unknown	224	275		71	79	
Grade				<0.001			0.24
	Well; I	412	146		67	60	
	Moderately; II	722	349		120	115	
	Poorly;III	169	168		40	57	
	Undifferentiated; IV	18	10		2	0	
	Unknown	693	704		186	183	
Tumor stage				<0.001			0.25
	T1	944	412		151	117	
	T2	429	317		84	29	
	T3	138	131		37	36	
	T4	81	92		18	100	
	TX	422	425		125	133	
Node status				<0.001			0.72
	N0	1168	344		154	163	
	N1	181	130		42	49	
	N2	294	454		103	103	

Variables	No Chemotherapy	Chemotherapy	P value	No Chemotherapy	Chemotherapy	P value
N3	57	184		21	20	
NX	314	265		95	80	
Metastasis status			<0.001			0.003
M0	1448	593		243	215	
M1	486	734		146	187	
MX	80	50		26	13	
Summary stage			<0.001			<0.001
Localized	852	97		111	56	
Regional	486	346		100	117	
Distance	614	907		183	233	
Unknown	62	27		21	9	
Surgery			<0.001	187	173	0.49
No	1249	414		227	241	
Yes	762	960				
Radiation			<0.001			0.87
No	1613	762		292	294	
Yes	401	615		123	121	

Abbreviation: PSM: Propensity-score matching; SD, standard deviation

Supplementary Table S3: Overall survival stratified by clinical characteristics for patients with Papillary Adenocarcinoma

Characteristics	3-year	95% CI	5-year	95% CI	10-year	95% CI
Total	0.43	(0.41-0.45)	0.32	(0.30-0.34)	0.22	(0.21-0.25)
Age						
<=78	0.44	(0.42-0.46)	0.33	(0.31-0.35)	0.23	(0.21-0.25)
>78	0.36	(0.31-0.42)	0.27	(0.22-0.33)	0.20	(0.14-0.28)
Gender						
Female	0.44	(0.42-0.47)	0.33	(0.3-0.35)	0.23	(0.21-0.26)
Male	0.42	(0.39-0.44)	0.31	(0.28-0.34)	0.17	(0.14-0.22)
Race						
White	0.43	(0.41-0.45)	0.32	(0.3-0.34)	0.23	(0.21-0.25)
Black	0.37	(0.31-0.43)	0.30	(0.25-0.37)	0.20	(0.15-0.28)
Other	0.45	(0.4-0.5)	0.34	(0.29-0.4)	0.21	(0.16-0.28)
Marital status						
Married	0.43	(0.41-0.46)	0.32	(0.29-0.34)	0.22	(0.2-0.25)
Single	0.42	(0.39-0.45)	0.32	(0.29-0.35)	0.22	(0.19-0.26)
Laterality						
Right	0.45	(0.43-0.48)	0.34	(0.32-0.37)	0.25	(0.22-0.28)
Left	0.43	(0.4-0.46)	0.32	(0.29-0.35)	0.23	(0.2-0.26)
Bilateral	0.15	(0.08-0.31)	0.05	(0.01-0.19)	/	
Lobe						
Main bronchus	0.19	(0.1-0.33)	0.15	(0.07-0.29)	0.12	(0.05-0.26)
Upper	0.45	(0.42-0.47)	0.35	(0.32-0.38)	0.26	(0.23-0.29)
Middle	0.58	(0.5-0.66)	0.47	(0.39-0.57)	0.31	(0.22-0.44)
Lower	0.51	(0.48-0.54)	0.38	(0.35-0.42)	0.25	(0.22-0.29)
Overlapping lesion of lung	0.36	(0.23-0.55)	0.21	(0.11-0.4)	0.17	(0.08-0.36)
Grade						
Well; I	0.64	(0.6-0.69)	0.53	(0.48-0.58)	0.35	(0.3-0.42)
Moderately; II	0.57	(0.54-0.6)	0.45	(0.41-0.49)	0.33	(0.29-0.37)
Poorly;III	0.38	(0.32-0.44)	0.26	(0.21-0.32)	0.19	(0.14-0.25)
Undifferentiated; IV	0.41	(0.25-0.66)	0.36	(0.21-0.62)	0.30	(0.16-0.57)
Tumor stage						
T1	0.60	(0.57-0.63)	0.51	(0.48-0.54)	0.41	(0.37-0.44)
T2	0.46	(0.43-0.51)	0.33	(0.29-0.37)	0.20	(0.16-0.25)
T3	0.36	(0.3-0.43)	0.23	(0.18-0.3)	0.12	(0.07-0.2)
T4	0.36	(0.29-0.45)	0.18	(0.13-0.27)	0.08	(0.04-0.16)
Tumor size						
<= 38 mm	0.58	(0.55-0.61)	0.49	(0.47-0.53)	0.38	(0.35-0.42)
> 38 mm	0.39	(0.36-0.43)	0.24	(0.20-0.27)	0.13	(0.10-0.17)
Node status						
N0	0.65	(0.63-0.68)	0.56	(0.53-0.59)	0.44	(0.4-0.48)
N1-3	0.29	(0.27-0.32)	0.17	(0.15-0.2)	0.09	(0.07-0.12)

Characteristics	3-year	95% CI	5-year	95% CI	10-year	95% CI
Metastasis status						
M0	0.58	(0.56-0.61)	0.48	(0.45-0.5)	0.35	(0.33-0.38)
M1	0.17	(0.15-0.2)	0.07	(0.05-0.09)	0.02	(0.01-0.04)
Summary stage						
Localized	0.77	(0.74-0.8)	0.69	(0.65-0.72)	0.57	(0.52-0.61)
Regional	0.52	(0.48-0.56)	0.37	(0.33-0.41)	0.22	(0.18-0.27)
Distance	0.17	(0.15-0.19)	0.07	(0.06-0.09)	0.03	(0.02-0.04)
TNM						
I	0.82	(0.8-0.85)	0.74	(0.71-0.78)	0.60	(0.55-0.65)
II	0.62	(0.57-0.68)	0.47	(0.41-0.54)	0.30	(0.24-0.38)
III	0.37	(0.32-0.43)	0.24	(0.2-0.29)	0.15	(0.11-0.21)
IV	0.17	(0.15-0.2)	0.07	(0.05-0.09)	0.02	(0.01-0.04)
Surgery						
Yes	0.69	(0.66-0.71)	0.57	(0.54-0.6)	0.41	(0.38-0.45)
No	0.17	(0.15-0.19)	0.07	(0.06-0.09)	0.04	(0.03-0.05)
Radiation						
No	0.50	(0.48-0.53)	0.40	(0.37-0.42)	0.30	(0.27-0.32)
Yes	0.26	(0.23-0.29)	0.14	(0.12-0.17)	0.07	(0.05-0.09)
Chemotherapy						
No	0.53	(0.5-0.55)	0.43	(0.4-0.46)	0.32	(0.3-0.35)
Yes	0.29	(0.27-0.32)	0.16	(0.14-0.19)	0.09	(0.07-0.12)

Abbreviation: 95% CI, 95% Confidence interval

Supplementary Table S4: Characteristics of patient with Papillary Adenocarcinoma and matched Common-Adenocarcinoma

Characteristics	Papillary Adenocarcinoma	Common-Adenocarcinoma	P-values
Total	3236	3236	
Age(Mean±SD)	66.4±11.3	66.5±11.2	0.93
Gender			0.78
Female	1720	1709	
Male	1516	1527	
Race			0.56
White	2533	2531	
Black	326	304	
Unknown	377	401	
Marital status			0.41
Married	1813	1780	
Single	1302	1316	
Unknown	121	140	
Lobe			0.63
Main bronchus	1442	1414	
Upper	155	161	
Middle	987	1042	
Lower	57	48	
Overlapping lesion of lung	37	31	
Unknown	558	540	
Grade			0.54
Well; I	423	380	
Moderately; II	975	1006	
Poorly;III	364	371	
Undifferentiated; IV	26	23	
Unknown	1451	1456	
Tumor stage			0.72
T1	1275	1238	
T2	666	690	
T3	246	261	
T4	152	165	
TX	897	882	
Node status			0.83
N0	1384	1360	
N1-3	1259	1279	
NX	593	597	
Metastasis status			0.97
M0	1828	1827	
M1	1269	1274	
MX	139	135	

Characteristics		Papillary Adenocarcinoma	Common-Adenocarcinoma	P-values
Surgery				0.77
	No	1362	1369	
	Yes	1866	1856	
	Unknown	8	11	
Radiatoin				0.23
	No	2277	2233	
	Yes	959	1003	
Chemotherapy				0.41
	No	1992	1959	
	Yes	1244	1277	

Abbreviation: SD, standard deviation
